# Human cognitive enhancement and reprogenetic technologies in Malaysia – A survey study of local Muslim undergraduate students' viewpoints

**DOI:** 10.3389/fsoc.2025.1701007

**Published:** 2026-01-15

**Authors:** Sayyed Mohamed Muhsin, Mohamed Aslam Akbar, Sohela Mustari, Mohammed H. Alashaikh, Alexis Heng Boon Chin

**Affiliations:** 1 Department of Fiqh and Usul al-Fiqh, AHAS KIRKHS, International Islamic University Malaysia, Gombak, Selangor, Malaysia; 2 Islamic Sciences Research Center, Imam Mohammad Ibn Saud Islamic University, Riyadh, Saudi Arabia; 3 Department of Economics, Kulliyyah of Economics and Management Sciences, International Islamic University Malaysia, Gombak, Selangor, Malaysia; 4 Department of Sociology and Anthropology, AHAS KIRKHS, International Islamic University Malaysia, Gombak, Selangor, Malaysia; 5 Singapore Fertility and IVF Consultancy Pvt Ltd., Singapore, Singapore

**Keywords:** brain microchip, brain-computer interface (BCI), fatwa, germline genome editing, Islam, PGT, reprogenetics, shariah

## Abstract

**Introduction:**

Newly emerging human enhancement technologies such as brain chip implants, CRISPR-Cas9-based gene editing, and polygenic embryo screening (PES) alongside preimplantation genetic testing (PGT-P) are highly controversial in Islam. However, the prevailing sociocultural dynamics encourage their uptake. In the current era of declining fertility rates, increased parental investment in fewer children has resulted in a flourishing tuition industry, accompanied by heightened academic pressure on students and widespread parental anxiety. These emerging technologies can be employed for cognitive enhancement, thereby providing an expedient solution for parents and students navigating a highly competitive educational environment.

**Materials and methods:**

To inform and facilitate future policy decision-making, an online survey was conducted among 575 undergraduate Muslim students at the International Islamic University Malaysia (IIUM) to assess their perspectives and opinions regarding these newly emerging technologies.

**Results:**

The findings indicated a significant level of opposition among respondents to the uptake of human enhancement technologies, with 54.8% opposing polygenic embryo screening, 69.2% opposing gene editing, and 75.3% opposing brain chip implants, reflecting substantial concerns about altering natural human attributes. The results also indicate that numerous Muslim respondents believe that Allah created humans flawlessly and purposefully, asserting that humanity lacks the authority to alter or amend this creation.

**Discussion/Conclusion:**

A three-pronged governance approach for human enhancement technologies is thus proposed, which encompasses (i) bioethical safeguards, (ii) public engagement and education, and (iii) economic accessibility. It is suggested that the Malaysian government should actively consult relevant stakeholders and various segments of the public before enacting future legislation on these technologies.

## Introduction

In recent years, Malaysia, similar to many other Asian nations, has witnessed a significant decrease in its fertility rate. In 2022, this rate reached a 50-year low of 1.6 children per woman, raising considerable alarm among Malaysian government officials and policymakers (The Sun, 2023; Mahari, 2023). Financial pressures stemming from inflation and the rising cost of living, worsened by the recent COVID pandemic, along with heightened work demands and the escalating expenses associated with child-rearing and education, have dissuaded many families from expanding their households in recent years (The Sun, 2023; Mahari, 2023).

Due to the current demographic trend of decreasing fertility rates, parents often channel all their hopes and dreams into a smaller number of children, allocating more financial and caregiving resources per child (Chin, 2024). Although this can bring about certain advantages, such as raising the human development index, parents often have heightened expectations and frequently pressurize their offspring to perform academically. In Malaysia, as in numerous Asian cultures, there is a pronounced cultural inclination for individuals to measure themselves against their peers, which often leads to personal dissatisfaction when they view others as more accomplished than themselves. This trend is particularly noticeable in parenting approaches. A notable example of this is the widespread use of private enrichment and tutoring programs (Chong, 2024). Many parents often feel obliged to invest significant financial resources in these programs, motivated by the fear that their children may fall behind academically compared to their peers if they do not engage in such activities.

Consequently, newly emerging innovative human enhancement technologies aimed at improving intelligence and academic performance are expected to experience significant demand in the near future, potentially leading to a profitable market in Malaysia and globally. These emerging technologies may offer an expedient solution for numerous prospective parents navigating an increasingly competitive educational landscape, especially those who can afford their high costs. These technologies encompass: (i) Polygenic embryo screening (PES) combined with preimplantation genetic testing (PGT-P) (Chin et al., 2024a), (ii) Germline genome editing utilizing CRISPR technology (Waltz et al., 2024), and (iii) Brain chip implants designed to enhance intelligence and memory (Chin et al., 2024b). At present, these newly emerging technologies have generated considerable ethical and moral debate, inciting vigorous discussions among medical doctors, biomedical scientists, lawmakers, religious leaders, and society as a whole (Chin et al., 2024a,b,c; Waltz et al., 2024; Chen, 2022; Rueda et al., 2022; Janssens, 2015).

At this juncture, it would be useful to briefly examine global attitudes toward human enhancement technologies, based on previous comparative ELSI (Ethics, Legal & Social Issues) studies. With regards to human gene editing, numerous survey studies have indicated that public opinion worldwide is overwhelmingly opposed to its application for human enhancement (even if safety concerns are to be sufficiently addressed in the future), but is much more receptive to its application for disease treatment and prevention (Taguchi et al., 2019; Watanabe et al., 2020; van Dijke et al., 2021; Kobayashi et al., 2022; Ramos et al., 2023). By contrast, survey studies have demonstrated that respondents are generally more receptive to the application of polygenic embryo screening for human enhancement, as compared to gene editing, because it does not result in any permanent man-made genetic modifications that can be passed down to future generations (Haining et al., 2025; Meyer et al., 2023). To date, there have not yet been any survey studies on public attitudes toward cognitive-enhancing brain chips due to its current nascent state of development. Previous Islamic bioethics literature have generally voiced a negative opinion on human enhancement technologies due to contravention of *Shari'ah* principles (Chin et al., 2024a; Isa et al., 2020; Chin et al., 2025a), but there have not yet been any survey study to gauge Muslim public attitudes toward these technologies. Hence, this study was conducted to fill the current gap in the academic literature.

In the face of such ethical and moral concerns, the pertinent question is whether Malaysia should embrace and permit such new technologies, given that these are expected to be heavily restricted or even banned by many developed countries worldwide. These raise serious ethical and social concerns, such as exacerbating inequality, enabling coerced or ill-informed use of risky technologies, and undermining local moral standards and family values concerning reproduction and parenting. The possible negative impacts must be thoroughly considered in relation to any economic advantages. Malaysia's emergence as a potential hub for ethically controversial human enhancement technologies is conceivable, given its track record as a destination for contentious assisted reproduction procedures. For instance, sex selection using the PGT-A technique is prohibited in many countries but is practiced with relative freedom in Malaysia, thereby attracting numerous foreign patients (Chin, 2022). Egg donors are scarce in many countries because payment is banned. By contrast, payment for egg donation is often overlooked in Malaysia, and non-Muslim egg donors are plentiful and easy to source (Romero, 2019; Tan, 2019).

Hence, this survey study was carried out to gauge Malaysian Muslim viewpoints and attitudes toward such newly emerging human cognitive enhancement and reprogenetic technologies, which could facilitate and inform future policy-making decisions on permitting or regulating their use in Malaysia. By analyzing the survey responses, this study assesses public perception, key concerns, and the potential societal impacts that these new technologies will have on the Muslim community in Malaysia, which currently constitute the majority of the country's population. The data obtained would thus enable us to better understand the Muslim majority's stance on the acceptability of such technologies in Malaysia, which could facilitate future bioethical discourses on these new technologies within the framework of Islamic *Shari'ah* law. Additionally, the results of this study will also inform and facilitate future government policy-making on regulating these newly emerging technologies.

## Materials and methods

The respondents for this study are 575 local Muslim undergraduate students of the International Islamic University of Malaysia (IIUM), who volunteered to participate at their own free will. The criteria for participation included being (i) an undergraduate student at the International Islamic University of Malaysia, (ii) of Malaysian nationality, and (iii) an adherent of Islam. All IIUM undergraduates are required to complete a set of university core courses offered by the Department of Fundamental and Inter-Disciplinary Studies (FIDS), such as Basic Philosophy and Islamic Worldview, Knowledge and Civilization in Islam, and Ethics and Fiqh of Contemporary Issues. These courses aim to provide a foundational understanding of Islamic epistemology, ethics, and jurisprudence as applied to modern contexts. Prior to completing the questionnaire, the students were also given a brief overview of human enhancement technologies and their potential ethical and legal implications in Islam to ensure that their responses reflected informed perspectives.

IIUM was chosen as the venue for this survey study, as the student body is predominantly Muslim, making them a suitable population for this study that sought to investigate how Islamic beliefs influence perception of bioethical issues. Moreover, IIUM promotes the integration of Islamic values with modern sciences, so IIUM students, as the study subjects, could provide a unique context for understanding how their Islamic religious background influences their attitudes toward newly emerging human enhancement technologies.

The “Introduction to the Survey Study” and the “Questionnaire Form” that were posted online for this study are attached as “Supplementary Information 1” and “Supplementary Information 2,” respectively. Both of these documents are bilingual, written in English and Malay. The specific framework employed for this survey study and development of the questionnaire form were mainly based on and adapted from two previous survey studies that were conducted in Singapore (Haining et al., 2025) and the USA (Meyer et al., 2023), with some in-house modifications that were inspired by our previous studies on the ethical aspects of human enhancement technologies such as gene editing (Chin et al., 2025b), polygenic embryo screening (Chin et al., 2024a,c) and cognitive-enhancing brain chips (Chin et al., 2024b, 2025a). The survey study was conducted online by utilizing Google Forms. The recruitment of student respondents was carried out through announcements by university lecturers during teaching sessions. A representative sample of 575 respondents participated in this survey, and their demographics are summarized in Table 1. Among them, 163 (28.35%) were Male and 412 (71.65%) were female. As the survey respondents were undergraduate students, all of them were below 25 years of age. The survey data were collected over a period of 2 months (January–March 2025). Respondents were asked to rate their agreement with various statements on a Likert scale: Strongly Agree, Agree, Neutral, Disagree, and Strongly Disagree. These responses were then quantitatively analyzed by calculating the frequency of each answer for every question, which provided insights into the general trends and opinions of local Muslim undergraduates regarding human enhancement technologies. Frequency distributions were used to illustrate the responses for each question, with further analysis being segmented by gender. A relationship test was conducted using the Pearson Chi-squared test to ascertain the relationship between categorical variables. The Chi-squared test was used as the variables are qualitative in nature. To evaluate the strength of associations, Cramer's V (for contingency tables larger than 2 × 2) and Phi coefficients (for 2 × 2 tables) were also computed. Ninety-five percent confidence intervals were reported to provide precision of these effect size estimates. All statistical analyses were performed using the SPSS (version 28) software. Within the questionnaire, survey respondents were asked to provide a written qualitative answer to enable a better understanding of their views in depth. The written responses in the questionnaire form were analyzed using a thematic analysis approach, following the six-phase framework proposed by Braun and Clarke (2006): familiarization with data, generation of initial codes, identification of themes, reviewing themes, defining and naming themes, and producing the final report. The data were coded manually by two independent researchers to enhance the reliability of theme identification. Any discrepancies in coding were discussed and resolved through consensus. Representative quotations were then selected to illustrate the key themes and subthemes that emerged from the analysis.

**Table 1:** Demographic background of survey respondents.

**Sample characteristics (*n* = 575)**	***N***	**%**
**Gender**		
Male	163	28.35
Female	412	71.65
**Age bands**		
Age < 25 years	575	100.00
**Highest qualification**		
Undergraduate	575	100.00

A limitation of this survey study is that it was conducted exclusively among undergraduate Muslim students at the International Islamic University of Malaysia (IIUM), an institution that integrates Islamic perspectives into its curriculum. Consequently, the views expressed by respondents may reflect a higher degree of religious awareness and alignment with Islamic principles compared to the broader Malaysian Muslim population. The findings should therefore not be generalized to all Malaysian Muslims or other demographic groups. Future studies should include participants from a wider range of universities and sociocultural contexts to capture more diverse perspectives.

## Results

**Figure 1:**
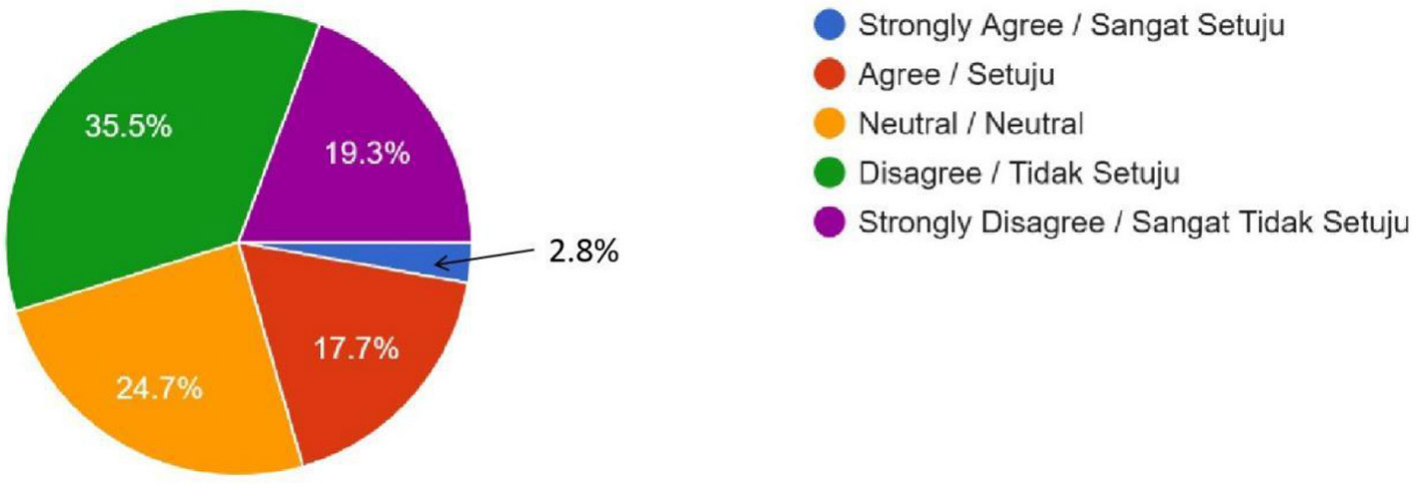
Embryo Screening (No Genetic Modification)—Do you feel it is OK to use DNA testing for choosing human IVF embryos based on traits like intelligence or physical appearance (height or skin color), even when there is no health reason to do so? (For example, selecting an embryo with genes for high IQ instead of just letting nature decide).

As illustrated in Table 2, Muslim public opinion on human-enhancement technologies was predominantly negative. Polygenic embryo screening attracted substantial opposition, with 204 respondents registering “Disagree” (35.5%) and a further 111 registering “Strongly Disagree” (19.3%), whereas only 118 respondents fell into the combined “Agree” and “Strongly Agree” (20.5%) strata (Figure 1). The qualitative response from this survey showed a mixed opinion on polygenic embryo screening. One of the female respondents from the Kulliyah (Faculty) of Education expressed her attitude towards embryo screening by saying, “All of us are the creation of Allah, and I believe Allah has made us for a reason. So, we do not need to screen earlier just to make our future better by going against Allah's will.” However, a different opinion came from a male student of the Kulliyah (Faculty) of Science, who praised the development of technology and stated that people should grab the benefit of this development. At the same time, he reminded all believers to be cautious in grabbing the benefit if it conflicts with religion. He stated, “Embryo screening is too advanced technologically, and we should accept it unless it is against our religion.”

**Table 2:** Frequency distribution of responses to survey questions.

**Question**	**Strongly agree**	**Agree**	**Neutral**	**Disagree**	**Strongly disagree**	***P-Value (Chi-Squared Test)***
Polygenic Embryo Screening (Is it acceptable to select embryos based on traits like intelligence or appearance?)	16 (2.8%)	102 (17.7%)	142 (24.7%)	204 (35.5%)	111 (19.3%)	–
Female	12	72	96	143	89	0.239
Male	4	30	46	61	22	
Gene Editing (Is it acceptable to edit genes for enhancing intelligence?)	11 (2%)	71 (12.3%)	95 (16.5%)	276 (48%)	122 (21.2%)	–
Female	5	43	63	210	91	0.014
Male	6	28	32	66	31	
Brain Chips (Is it acceptable to use brain chip implants for enhancing memory or intelligence in healthy individuals?)	9 (1.5%)	49 (8.5%)	84 (14.5%)	246 (42.9%)	187 (32.6%)	–
Female	4	33	56	182	137	0.287
Male	5	16	28	64	50	
Religious Concerns (Should the government ban technologies like gene editing for Muslims due to *Shari'ah* concerns?)	157 (27.3%)	221 (38.4%)	89 (15.5%)	74 (12.9%)	34 (5.9%)	–
Female	120	158	54	53	27	0.089
Male	37	63	35	21	7	
Social Inequalities (Do you think that uptake of these human enhancement technologies will increase social inequalities, because only the rich can afford these?)	268 (46.6%)	219 (38.1%)	63 (11%)	21 (7.6%)	4 (0.7%)	–
Female	193	153	43	19	4	0.202
Male	75	66	20	2	0	
Racial Disparities (Do you expect that there will be disparities in the uptake of these human enhancement technologies by the different races and ethnic groups in Malaysia, which might widen the socioeconomic gap between the different races?)	167 (29%)	242 (42.1%)	121 (21%)	42 (7.3%)	3 (0.6%)	–
Female	114	175	92	28	3	0.428
Male	53	67	29	14	0	
Legal Approval (Do you think that the Malaysian government should approve and permit the uptake of these human enhancement technologies in the country?)	25 (4.3%)	60 (10.4%)	176 (30.6%)	186 (32.3%)	128 (22.3%)	–
Female	13	45	120	134	100	0.067
Male	12	15	56	52	28	
Permitting or banning according to religion (Do you think that it would be fair for the Malaysian government to enact different laws and regulations governing the uptake of human enhancement technologies by Muslim vs. non-Muslim patients)?	176 (30.6%)	195 (33.9%)	96 (16.7%)	70 (12.2%)	38 (6.6%)	–
Female	136	130	60	57	29	0.012
Male	40	65	36	13	9	
Public Subsidies (Should there be public subsidies for the poor to access these technologies?)	115 (20%)	215 (37.4%)	138 (24%)	67 (11.7%)	40 (6.9%)	–
Female	77	156	102	48	29	0.791
Male	38	59	36	19	11	
Social Pressure (Would you feel social pressure to use these technologies if others around you do?)	69 (12%)	148 (25.7%)	149 (25.9%)	139 (24.2%)	70 (12.2%)	–
Female	47	103	104	110	48	0.273
Male	22	45	45	29	22	
Financial Burden (Would the high costs of these technologies influence your decision to have fewer children?)	83 (14.4%)	178 (31%)	171 (29.7%)	101 (17.6%)	42 (7.3%)	–
Female	63	135	117	74	23	0.057
Male	20	43	54	27	19	

**Figure 2:**
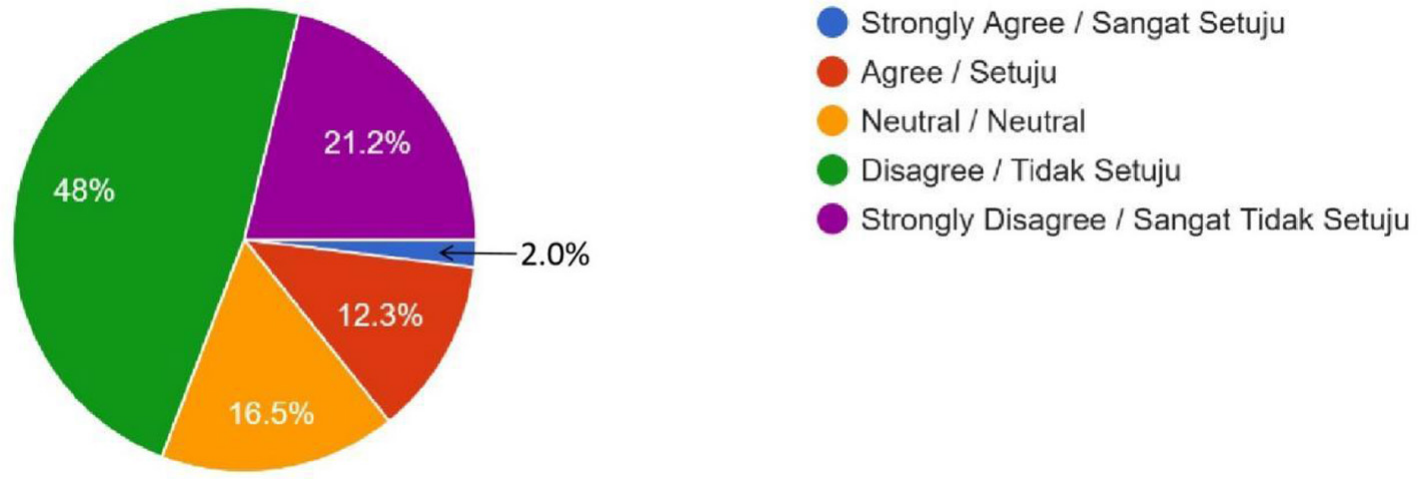
Gene Editing—Do you think it is OK to change an embryo's genes to improve traits like intelligence, knowing these changes will be passed on to future generations? (For example, editing genes to make a child smarter, which would affect their future children as well).

**Figure 3:**
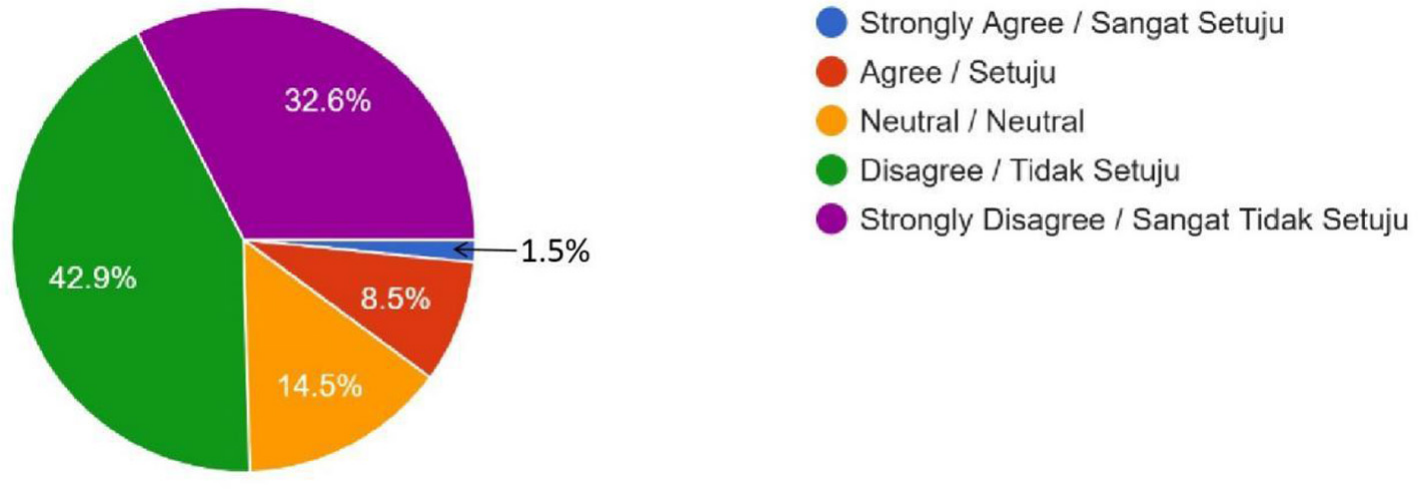
Brain Chips—Do you think it is OK to use brain implants like a microchip to make someone smarter, even if they don't have any medical problems? (For example, implanting a chip to enhance memory or learning skills in a healthy person).

**Figure 4:**
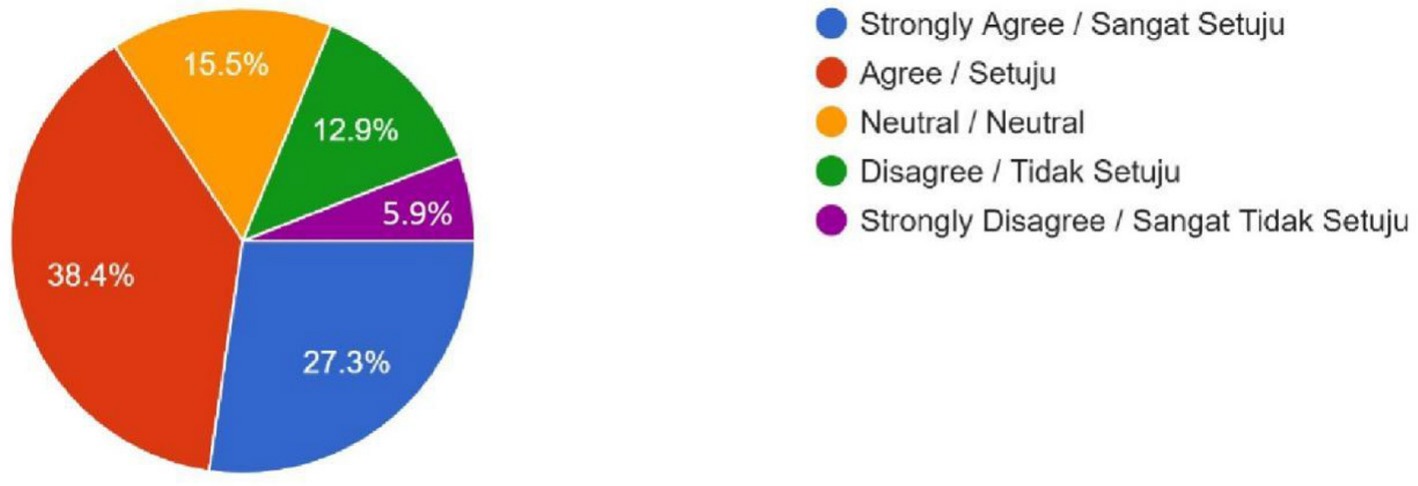
Religious Concerns—Should the government ban technologies like gene editing for Muslims due to *Shari'ah* concerns?

**Figure 5:**
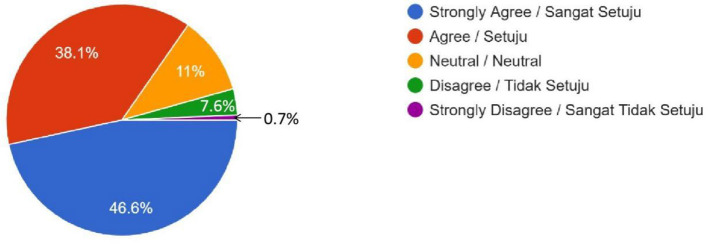
Social Inequalities—Do you think that uptake of these human enhancement technologies will increase social inequalities, because only the rich can afford these? Will this further widen the gap between the rich and the poor?

**Figure 6:**
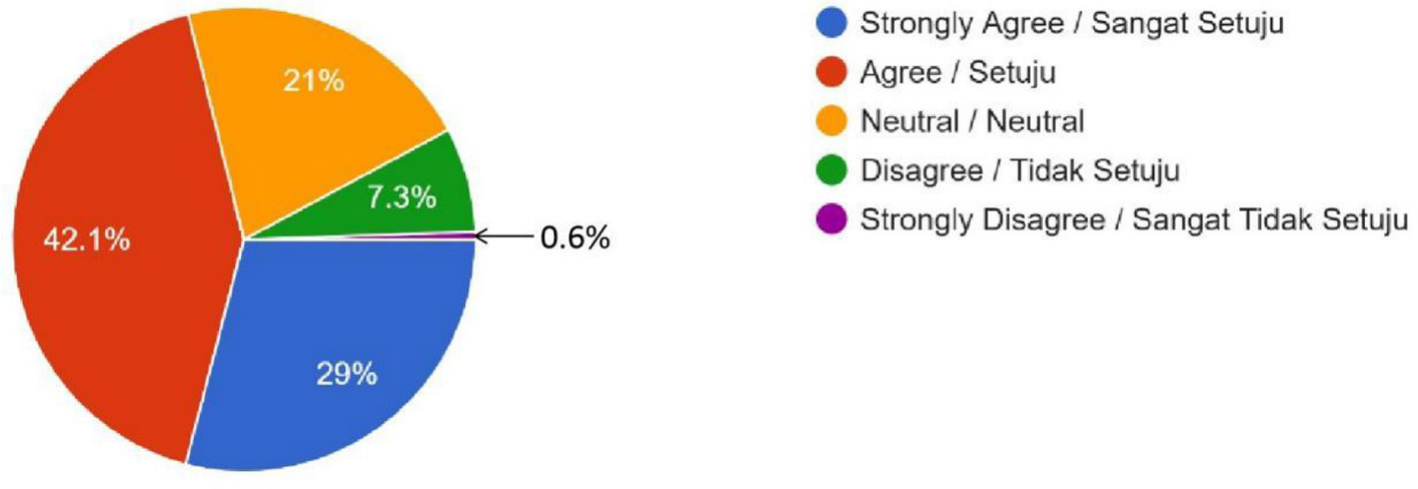
Racial Disparities—Do you expect that there will be disparities in the uptake of these human enhancement technologies by the different races and ethnic groups in Malaysia, which might widen the socioeconomic gap between the different races?

**Figure 7:**
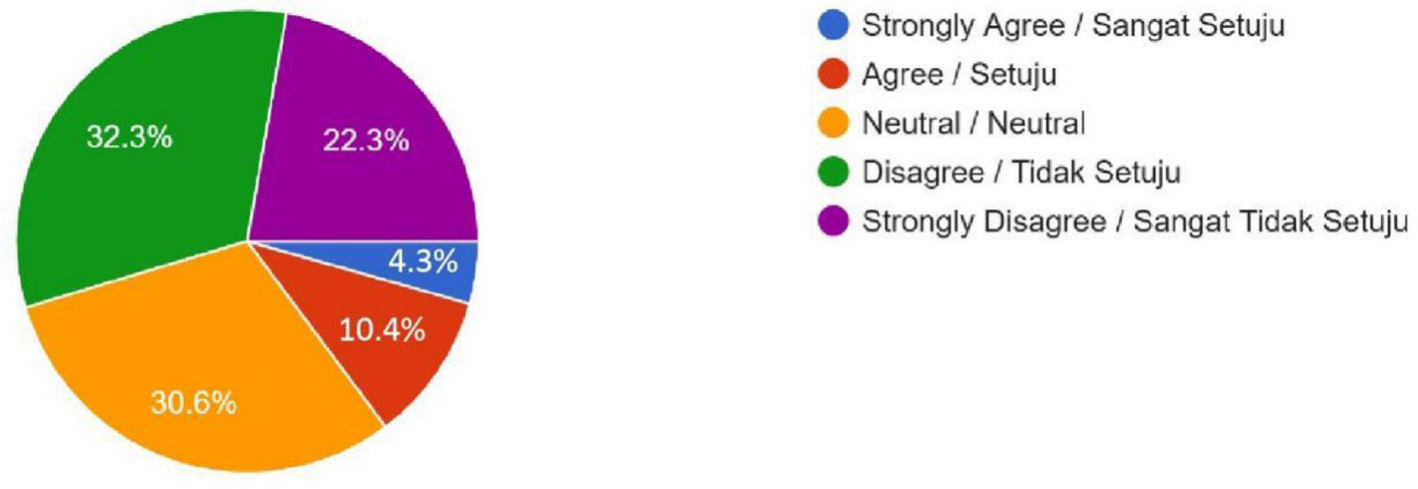
Legal Approval—Do you think that the Malaysian government should approve and permit the uptake of these human enhancement technologies in the country?

**Figure 8:**
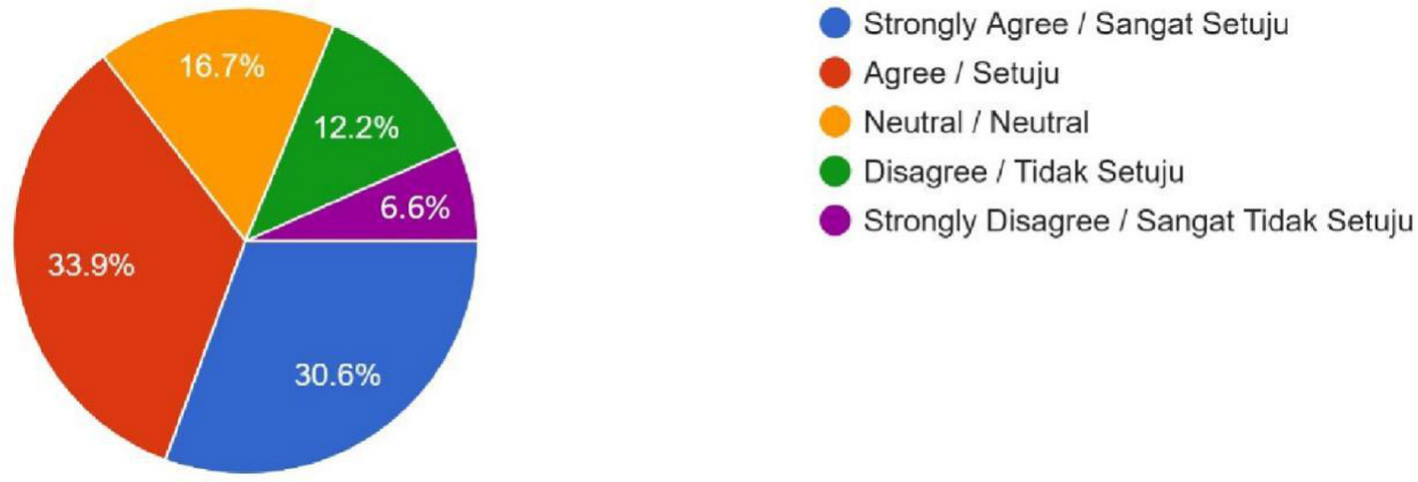
Permitting or banning according to religion—Do you think that it would be fair for the Malaysian government to ban the uptake of some of these human enhancement technologies by Muslims due to violation of *Shari'ah* law (for example, gene editing for enhancement would be alteration of Allah's creation), while permitting uptake by non-Muslims?

The religious concerns regarding the permissibility of gene editing and brain chip implants in Islam were even more tilted toward disapproval. Many respondents strongly agreed that gene editing for enhancement would conflict with *Shari'ah* principles, which emphasize the sanctity of Allah's creation. These religious considerations align with broader concerns about Islamic injunctions on the limits of human intervention in natural processes. The survey data shows that gene editing was met with stronger opposition compared to polygenic embryo screening, registering 276 (48%) “Disagree” and 122 (21.2%) “Strongly Disagree” responses, dwarfing the remaining 177 (30.8%) respondents who expressed any level of agreement (Figure 2). The quantitative results of gene editing are also supported by the qualitative result. One of the male students from the Kulliyyah (Faculty) of Economics expressed the reason behind his attitude, “It is ethically contradictory with social and religious norms. God created humans as perfect; humans do not have the power to create humans. Human enhancement technologies raise concerns about interfering with the divine will by reducing human value and creating unfair advantages due to unequal access.” A similar response was expressed by a female student of the Kulliyyah (Faculty) of Islamic Revealed Knowledge and Human Sciences. She uttered that she did not have any hatred for technological development unless such technologies intend to challenge her creator, Allah (SWT). She also stated confidently, “In the Holy Qur'an, Allah (SWT) has forbidden any action that changes His creation. The Prophet (SAW) has also prohibited the same things because they are included in the category of changing the creation of Allah (SWT).” Interestingly, brain chip implants elicited even much stronger opposition that either polygenic embryo screening or gene editing, with the survey data showing that brain chip implants registered 246 (42.9%) “Disagree” and 189 (32.6%) “Strongly Disagree” responses, completely dwarfing the remaining 142 (24.5%) respondents who expressed any level of agreement (Figure 3). A female respondent from the Kulliyyah (Faculty) of Engineering voiced her view why she disagreed about brain chips. She stated, “We want the human being to experience the real life and human *fitrah* (natural disposition of mankind). However, it will harm the human being and will change the nature of life, and thus I am against the proposed brain chip.” A majority of 378 respondents (65.7%) agreed with the government prohibiting the uptake of human enhancement technologies by Malaysian Muslims due to conflict with Islamic principles (Figure 4), while an overwhelming 487 (84.7%) and 409 (71.1%) of respondents felt that the uptake of such technologies in the country would lead to social inequalities and racial disparities, respectively (Figures 5, 6). Overall, only 85 respondents (14.7%) agreed with the Malaysian government permitting the uptake of these human enhancement technologies in the country, as opposed to 314 respondents (54.6%) who disagreed (Figure 7). Nevertheless, a clear majority of 371 (64.5%) respondents agreed to having different laws and regulations governing the uptake of these new technologies by Muslim vs. non-Muslim patients (Figure 8).

**Figure 9:**
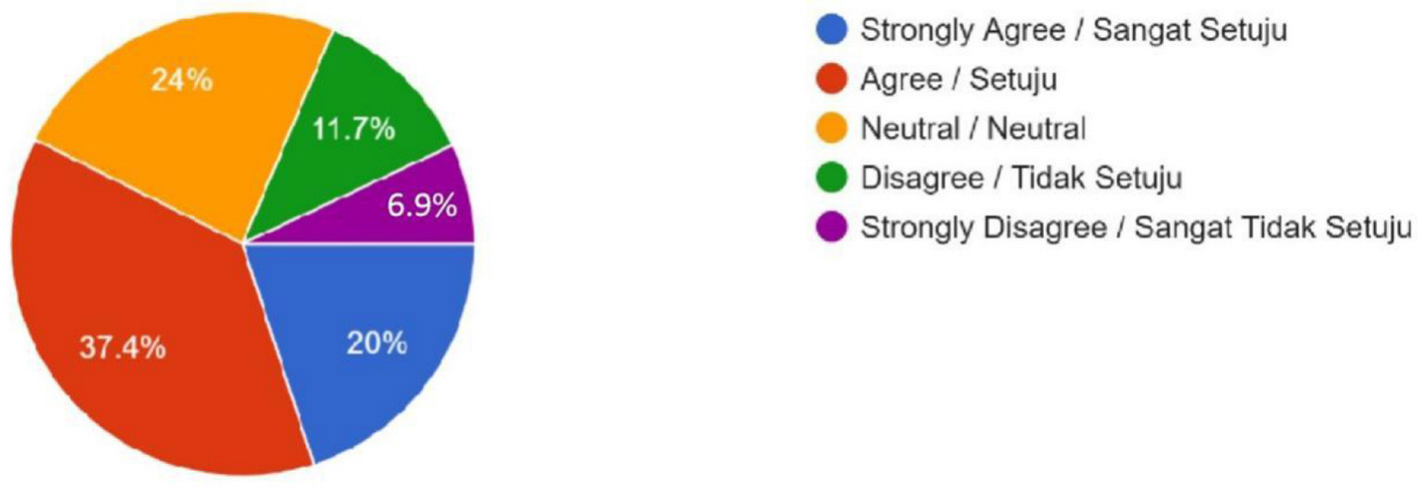
Public Subsidies—If the Malaysian government legally permits the uptake of these human enhancement technologies, should there be public healthcare subsidies for poorer people to utilize these technologies?

**Figure 10:**
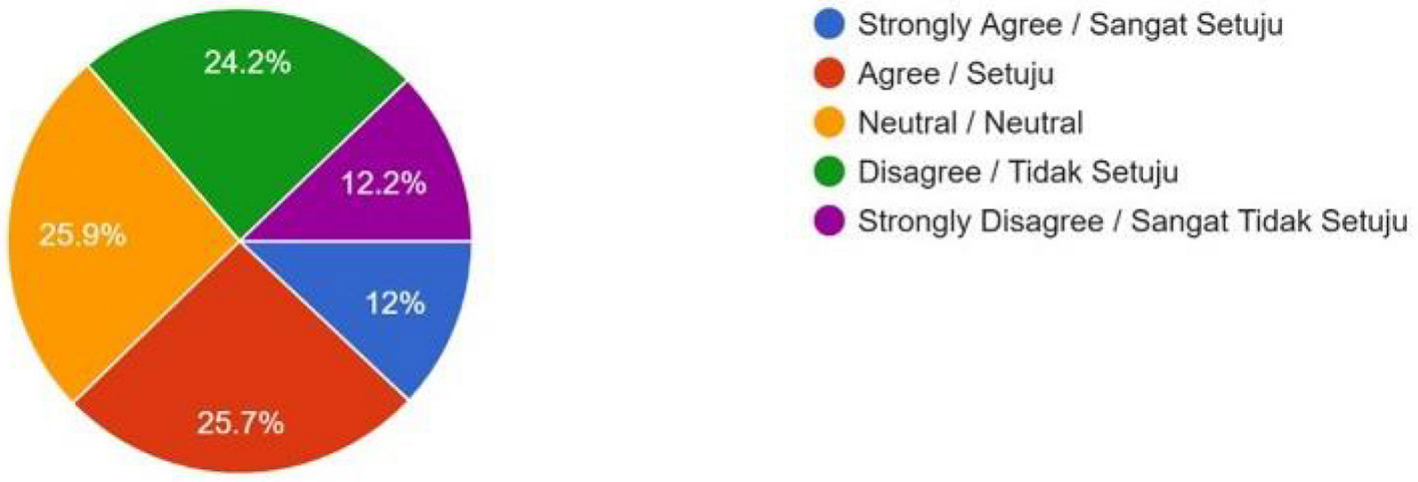
Social Pressure—If you see some of your friends and relatives using such human enhancement technologies to have smarter and more beautiful kids, will you be under social pressure to do the same?

**Figure 11:**
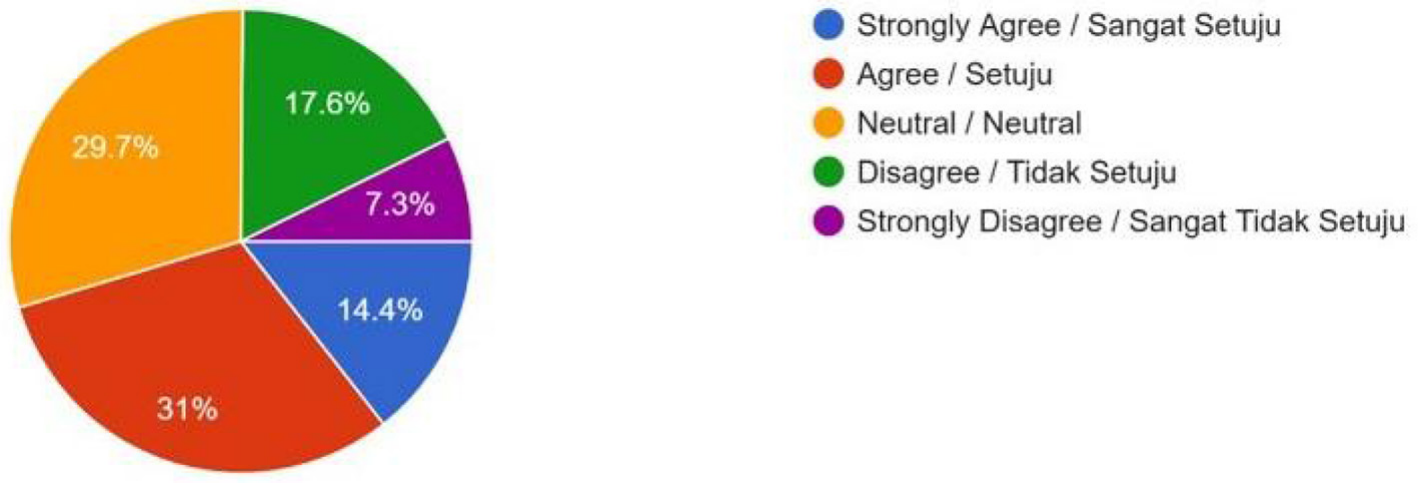
Fewer Children due to Financial Burden—If you feel obliged to spend so much money on such human enhancement technologies for your kids, will you plan to have fewer kids?

Despite overall general disapproval of human enhancement technologies, the majority of 330 (57.4%) respondents supported public subsidies for lower-income groups (Figure 9), if such technologies were to be legally permitted, which indicated that fair access is a core consideration in public acceptance among Muslims. Nevertheless, 107 (18.6%) respondents disagreed on giving public subsidies to the poor to access these technologies (Figure 9). To them, the subsidy may increase the possibility of going against the religious faith of Muslims. One of the female students from the Kulliyyah (Faculty) of Islamic Revealed Knowledge and Human Sciences stated, “Yes, giving subsidies may narrow the gap between the rich and poor in enjoying technological development, but still I do not support it. Because I believe that altering human biology is akin to playing God, disrupting the natural order, and potentially causing unintended consequences. As the poor people do not have proper education, they may be derailed from the Islamic ethos.” A significant number (24%) of respondents remained neutral as they are confused about public subsidies to help the poor to access human enhancement technologies (Figure 9). According to them, it is not religiously forbidden (*haram*) to have technological support if the person has any health issues. However, they remained cautious of expensive medical fees and inadvertent side effects of treatment with such advanced medical technologies. One undergraduate female student from the Kulliyah (Faculty) of Science stated, “It is not popular yet, and among the Muslims of Malaysia, many of them do not know the side effects. If the technology is used wrongly, it will cause health problems, creating unnatural processes and probable harm to future generations. We need a widespread awareness programme before mass utilization.” Only a minority of 217 (37.7%) respondents felt that social pressure would be a significant factor in influencing their uptake of human enhancement technologies (Figure 10), while a minority of 261 (45.4%) respondents felt that the high costs of taking up these technologies would discourage them from having more children (Figure 11).

Based on the written responses, a thematic analysis was performed on the Muslim undergraduates' viewpoints on human enhancement technologies. Several religious and ethical objections were raised against human enhancement via brain chips and reprogenetic technologies. The core of these objections often centers on the belief that such interventions interfere with divine will and the natural order of creation. A predominant objection is that these technologies involve changing Allah's (God's) creation. This is viewed as an “overreach of human authority” and “playing God.” Most of the survey respondents believe that Allah created humans perfectly and for a reason, and there is no need to change this. Modifying these creations implies dissatisfaction with God's design or that “Allah's creation is bad.” Such human enhancement technologies are thus seen as contradicting divine decree (*qaḍā'*) and predestination (*qadar*), and a lack of gratitude or belief in Allah's plan. Indeed, many of the survey respondents quoted verses from the Qur'an and *Hadiths* that prohibited changing Allah's creation without urgent need. For example, Surah An-Nisa' (Quran, 4:119) is often quoted, whereby Satan promises to command humans to “change the creation of Allah.” This verse implies that altering God's creation is a satanic act. Another Quranic verse from Surah At-Tin (Quran, 95:4), “Indeed, We created man in the best stature,” is referenced to highlight that Allah has already created humans in their “best form” or “perfectly.” Therefore, there is no need to change it. The Quranic verse from Surah Ar-Rum (Quran, 30:30), “The nature of Allah upon which He created mankind. There is no change in the creation of Allah,” is quoted to emphasize that changing Allah's creation is against the divine nature (*fiṭrah*) upon which humanity was created. Additionally, Surah Al-Imran (Quran, 3:191) is also mentioned to suggest reflecting on creation and acknowledging that God “did not create this in vain,” which implies accepting what God has created. *Hadith* narrations quoted by survey respondents often included those involving Prophet Muhammad (peace be upon him) prohibiting “changing the creation of Allah.” Specific *Hadiths* are mentioned that curse individuals who alter their appearance for beauty without medical need, such as those who tattoo, get tattoos, shave eyebrows, or file teeth, because these actions are considered “changing Allah's creation.” These *Hadiths* suggest that similar prohibitions would apply to human enhancement technologies. Overall, the core objections from the survey respondents were based on injunctions from both the Quran and *Hadiths*, which viewed human enhancement, especially for non-medical reasons, as interfering with the divine will, challenging and usurping Allah's role as Creator, and being ungrateful for His perfect creation. This also extends to the belief that it goes against divine decree (*qaḍā'*) and predestination (*qadar*). These sources repeatedly stress that changes to the human mind and body are only permissible for “dire need,” “emergency,” or “medical purposes” like illness or disfigurement.

Besides objections based on Islamic religious grounds, the survey respondents also voiced objections based on ethical, societal, and safety concerns. The most prominent among these are issues related to social inequality and injustice, with many respondents expressing concern that these technologies would widen the gap between the rich and the poor, potentially resulting in a “stratified society” and phenomena of “super humans and normal humans,” leading to “unfair advantages.” Some expressed concern about stigmatization or discrimination against those who are “non-enhanced” or have “undesirable traits.” Another area of concern is issues related to the safety and unknown risks of human enhancement technologies. Many respondents expressed fears about potential harm to individual health. They highlighted the lack of guaranteed success and the unknown long-term effects on individuals and future generations. This includes risks of “new diseases or disabilities,” as well as “hereditary disorders.” Then, there are ethical concerns related to the loss of human nature, identity, and authenticity. Many respondents believe these technologies go against “human nature.” There are fears of diminishing human dignity, reducing human value, blurring the line between humans and machines, and the potential loss of personal identity and individuality. Customizing children is seen as making family-building “less human.” Additionally, concerns also exist that human enhancement technologies could be misused for negative intentions, potentially leading to “moral decay” or even “mind control or body control,” as in the case of “cognitive-enhancing” brain chips. There are also concerns that humans would become overly dependent on technology and less motivated to achieve things through effort. While generally objected to, many responses indicate that these technologies might be permissible for health purposes only. This includes reasons such as “sickness,” “illness,” “disfigurement,” or “to stop hereditary diseases from continuing.” However, interventions for non-medical reasons, such as for “beauty” or “perfection,” or “simply to have a child to be smarter or brighter,” are generally deemed impermissible by the overwhelming majority of respondents. A small minority of opinions suggest a broader, albeit conditional, acceptance or even a positive outlook, with a few stating that if there are “no complications for the child after the change, then it's okay,” provided it follows Islamic law. The perspective that humans have their “own right to think” and “choose what they desire” is also mentioned by a few respondents, potentially supporting individual choice in enhancement.

Another key aspect of this study is to evaluate whether there are gender-related differences in Muslim perspectives on human enhancement technologies. The Chi-squared analysis data of *p* = 0.239 showed that there is no significant difference in gender (male vs. female) attitudes toward polygenic embryo screening for traits such as intelligence and appearance. Further Chi-squared analyses also showed no statistically significant differences in gender attitudes toward brain chip implants (*p* = 0.287), social inequalities (*p* = 0.202), racial disparities (*p* = 0.428), public subsidies (*p* = 0.791), social pressures (*p* = 0.273), and financial burdens (*p* = 0.057). However, the result showed that there is a statistically significant difference in gender attitudes toward gene editing for enhancing intelligence, as well as on whether the Malaysian government should enact different laws and regulations governing the uptake of these new technologies by Muslim vs. non-Muslim patients. The results thus suggest that Muslim men and women differ in their acceptance of gene editing for cognitive enhancement. Besides the Chi-square tests, effect size analyses using Cramer's V and Phi coefficients indicated that the observed associations between gender and attitudes toward the different human enhancement technologies were generally weak to moderate in magnitude (Cramer's V values ranging between 0.07 and 0.18), suggesting that while statistically significant differences were found in certain variables such as gene editing and legal approval (*p* < 0.05), the strength of association remained modest. This finding aligns with the notion that both male and female respondents share broadly similar ethical and religious outlooks, with only minor differences in their acceptance levels. Taken together, these quantitative findings provide a basis for further examining whether gender influences Muslim perspectives on human enhancement technologies.

Additionally, the qualitative results based on written responses also showed that men tend to display slightly higher acceptance of technological interventions, particularly when framed as progress or innovation. It is posited that women, being the primary carriers of offspring affected by technological changes, would be expected to display a more cautious attitude towards irreversible interventions in human biology such as gene editing. They would be expected to be more concerned about ethical risks, sociocultural consequences, and even the physical side effects of such new technologies. As the primary gatekeepers within families and societies, women are anticipated to be more in support of *Shari'ah*-based restrictions on such human enhancement technologies. However, in Malaysia, as the result suggests, Muslims of both genders are generally very much influenced by Islamic bioethics. The majority support genetic interventions for medical purposes only.

The written responses provided by Muslim female and male undergraduates showed a remarkable consistency in the core religious and ethical objections raised against human enhancement technologies by both genders, which overwhelmingly emphasize the prohibition of “altering Allah's creation” as the primary concern. While the fundamental objections are shared, some specific Quranic verses (e.g., 4:119, 95:4, 30:30) were explicitly cited by female respondents, while *Hadiths* prohibiting certain cosmetic alterations were explicitly mentioned by male respondents. However, the underlying principle derived from these texts—the impermissibility of changing Allah's creation for non-medical reasons—is consistently expressed by both sexes. They consistently view human enhancement as mankind attempting to overstep their boundaries and “play God” by interfering with divine design and creation, thereby violating the sanctity of Allah's creation and interfering with divine will. A major concern that was consistently expressed by both genders is the potential loss of human authenticity, identity, and nature with human enhancement technologies, diminishing what it means to be human. Some female responses explicitly mention the concern of people becoming like “robots” through human enhancement technologies. Overall, with regard to the written responses, there are no substantial differences in the types of religious and ethical objections raised by male and female undergraduates in the provided data. Both groups articulate very similar concerns, rooted deeply in Islamic principles regarding the sanctity of God's creation, divine will, and human nature.

## Discussion

In recent years, human enhancement technologies, including polygenic embryo screening, gene editing, and brain chip implants, have sparked heated debates globally. These technologies offer the potential for improving human traits such as intelligence, physical appearance, and even memory through artificial means. However, the ethical, social, and religious implications of these technologies remain contentious, particularly in societies with deep-rooted cultural and religious beliefs. This study thus explored Muslim public opinion on the acceptance of human enhancement and reprogenetic technologies in Malaysia, with a particular focus on polygenic embryo screening, gene editing, brain chip implants, and public healthcare subsidies for these technologies. It must be noted that the survey findings do not seek to determine the Islamic legal rulings on these technologies but rather to capture the prevailing ethical and religious perceptions among Muslim undergraduate students, which may inform broader discussions and policy deliberations. Regardless of the survey's findings, it cannot render permissible a technology that is prohibited under Islamic *Shari'ah* law, nor can it prohibit one that is permitted.

The survey findings reflect a Malaysian Muslim public that approaches human enhancement technologies with cautious skepticism, especially in non-therapeutic applications such as embryo screening, germline gene editing, and cognitive enhancement via brain chips. The high levels of disapproval with polygenic embryo screening (54.8%), gene editing (69.2%), and brain chip implants (75.5%) would suggest strong societal concern over the manipulation of human traits not grounded in medical necessity. These sentiments reinforce previous survey findings (Taguchi et al., 2019; Watanabe et al., 2020; van Dijke et al., 2021; Kobayashi et al., 2022; Ramos et al., 2023), which reported that unease about genome editing is primarily rooted in the ethical implications of altering what many consider to be the “essence” of humanity (Joseph et al., 2022).

Interestingly, despite such ethical reservations, 330 (57.4%) respondents either “strongly agreed” or “agreed” that public subsidies should be provided to make these technologies accessible to lower-income groups. This suggests a dual dynamic: while Malaysians may not fully endorse enhancement technologies, they acknowledge that if such technologies are permitted, they must be equitably accessed to avoid social injustice, thus indicating that fair access is a core consideration in public acceptance among Muslims. This resonates with the distributive justice principle outlined in the [UNESCO Declaration on Bioethics and Human Rights (UNESCO, 2005)], which emphasizes that advancement in biotechnology must not exacerbate social inequalities.

Moreover, the influence of social pressure—where 217 (37.7%) respondents acknowledged that they might feel compelled to adopt human enhancement technologies if others around them did—reflects Bauman's theory of liquid modernity (Bauman, 2000). In such a society, the pursuit of social approval often leads to ethical compromise, especially as technological progress redefines the norms of desirability and competitiveness (Bauman, 2000). This raises serious concerns regarding the freedom of individual choice and autonomy in a rapidly evolving techno-social environment.

As previously mentioned, female respondents demonstrated greater skepticism across multiple categories, especially concerning polygenic embryo screening and gene editing. This aligns with prior studies showing that women are generally more cautious about biotechnological interventions, possibly due to deeper socialization in care-giving roles and heightened awareness of long-term familial impacts (McGee, 2013). The gender gap suggests the importance of including female voices in bioethical policymaking, especially on issues relating to reproduction and family planning.

A notable dimension of this study is the religious dimension of ethical concern. More than half of all respondents either agreed or strongly agreed that such technologies should be banned for Muslims if they contradict *Shari'ah* principles. This may point to the need for dual-path regulation: one that respects religious diversity while also aligning with national bioethical frameworks. Islamic jurisprudence (*fiqh*) generally treats human germline genome editing with caution, primarily under the maxims of *la ḍarār wa lā ḍirār* (no harm nor reciprocating harm) and *al-ḍarurah tubiḥ al-maḥẓurāt* (necessity permits the prohibited). The principle of ḥ*ifẓ al-nasl* (protection of progeny), one of the five objectives of *Maqāṣid al-Sharī‘ah*, further complicates the permissibility of such interventions unless proven safe, necessary, and publicly beneficial. Thus, any future policy should include Islamic bioethics review panels to address theological concerns and build cross-sectarian consensus, particularly in a pluralistic society like Malaysia.

From an Islamic ethical standpoint, the participants' emphasis on maintaining Allah's creation, prohibiting its alteration, and preserving the *fiṭrah* (innate human nature) and the sanctity of the natural order reflects a theological conviction that human creation should not be subject to arbitrary modification. This reasoning parallels certain Western bioethical concerns grounded in the notions of human dignity and naturalness, which similarly caution against enhancement technologies that extend beyond therapeutic purposes. However, while secular bioethics often frames such arguments within the discourse of human rights or existential authenticity, Islamic ethics grounds them in divine will (*irādah ilāhiyyah*), *qaḍā'* and *qadar* (divine providence and predestination), human stewardship (*khilāfah*) as a trust rather than ownership over one's body, accountability before God, and the principle of preserving the integrity of God's creation (ḥ*ifẓ khalq Allāh*). These ethical foundations are further reinforced by the objectives of Islamic law (*maqāṣid al-sharī‘ah*) and detailed juristic deliberations derived from the *Qur'ān* and *Hadiths*.

One aspect that was overlooked in this survey study is how human enhancement technologies relate to the classical secular bioethics frameworks proposed by Beauchamp and Childress (2019), which is based on the four canonical principles of beneficence, non-maleficience, autonomy, and justice. At this juncture, it would be useful to compare and contrast how this secular bioethics framework relates to human enhancement technologies vs. the Islamic bioethics framework, which would reveal areas of both shared concern and foundational divergence. A fundamental divergence exists in that secular bioethics often permits individuals to take calculated risks in the pursuit of enhancement (balancing the four principles), whereas Islamic bioethics generally restricts such interventions to therapeutic necessity, guided by adherence to *Shari'ah* principles.

With regards to the principle of beneficence, secular proponents often appeal to “procreative beneficence,” arguing that parents have a moral responsibility to select the child most likely to achieve optimal well-being, endowed with the greatest advantages (Savulescu, 2001). Cognitive enhancement, for example, is argued to potentially enhance the autonomy of the offspring by improving reasoning ability (Schaefer et al., 2014). By contrast, Islamic bioethics aligns enhancement technologies with beneficence only when used therapeutically to protect life (ḥ*ifẓ al-nafs*), progeny (ḥ*ifẓ al-nasl*), and health (ḥ*ifẕ al-ṣiḥḥah*), based on the principle of necessity (ḍ*arurah*). The pursuit of enhancement for worldly perfection or competitive advantage is cautioned against, as it may be viewed as misplaced priorities over spiritual growth and devotion, as well as a lack of contentment with divine decree (*qadar*) (Ibrahim et al., 2019).

With regards to the principle of non-maleficence that requires avoiding harm, this may be challenged by the significant, known risks of some human enhancement technologies. For example, the on-target/off-target errors, mosaicism, and potential cancer risks associated with germline gene editing (Zischewski et al., 2017; Mehravar et al., 2019; Geng et al., 2023). The risks of using germline gene editing for mere enhancement are considered disproportionate to its potential benefits, thereby transgressing the secular principle of proportionality (Cwik, 2019). Another example is the unnecessary utilization of invasive procedures like IVF by healthy and fertile couples just for the sake of selecting embryos for non-disease traits such as intelligence via PES, which may be considered a form of clinical malpractice (Chin et al., 2024c). In a similar vein, Islamic jurisprudence aligns with non-maleficence by prioritizing the avoidance of harm over seeking potential benefits, emphasized by the legal maxims *al-darar yuzāl* (harm must be eliminated) and *lā ḍarar wa lā ḍirār* (no harm and no reciprocating harm) (Muhsin et al., 2019). This framework employs the precautionary principle of *sadd al-dharā'i‘* (blocking the means to harm) strictly (Muhsin et al., 2019), especially for non-therapeutic procedures, citing concerns about irreversible, heritable effects on future generations. There is a strong convergence of both frameworks, for example, shared concerns regarding the safety risks of brain chips (e.g., permanent brain damage or atrophy of natural functions) and gene editing, requiring strict safety measures (Muhsin et al., 2025). Nevertheless, there is a slight divergence here, with Islamic bioethics adopting a more stringent precautionary approach for enhancement technologies (*sadd al-dharā'i*) compared to secular bioethics, which often allows for calculated risks in pursuit of advancement (Muhsin et al., 2025).

With regards to the principle of autonomy, the secular bioethics framework often emphasizes individual reproductive autonomy (the parents' right to choose traits) (Savulescu, 2001). However, the key issue of contention is the offspring's lack of autonomy and free will (Bennett, 2009), as parents make all decisions about their future genetic makeup in reprogenetic technologies such as gene editing and PES (Chin et al., 2024c). With regards to the implantation of cognitive-enhancing brain chips, there are concerns that minors do not have the legal capacity to make such a major life-altering decision in the absence of any pressing medical need (Chin et al., 2024b, 2025a). Furthermore, there are also concerns that children and adolescents might be subjected to coercive pressure by their parents to take up brain chip implants (Chin et al., 2024b, 2025a). In a similar vein, the Islamic bioethics framework is also concerned about autonomy and legal capacity (*ahliyyah*) (Catic, 2023). Brain chip implants for minors are likely to be forbidden because minors are deemed to lack the cognitive maturity to make informed decisions (Catic, 2023). Likewise, reprogenetic enhancement through PES and gene editing is forbidden not only because these trespass on offspring autonomy, but also because these represent alteration to Allah's creation (*taghyir khalq Allāh*), which is a transgression against divine design (Fadel, 2010). Hence, both the secular and Islamic bioethics frameworks agree that minors should not be subjected to elective, risky procedures like brain chip implantation due to their limited cognitive maturity and consequent lack of legal capacity to make such life-altering medical decisions (Chin et al., 2024b, 2025a). Nevertheless, with regards to reprogenetic enhancement, secular bioethics debates whether parental autonomy overrides offspring autonomy (Savulescu, 2001; Bennett, 2009), whereas Islamic bioethics subordinates individual autonomy to the fundamental prohibition of altering Allah's creation (*taghyir khalq Allāh*) (Fadel, 2010).

With regards to the principle of justice (distributive justice), the secular perspective emphasizes social equity and the fair distribution of the benefits of these technologies. Secular bioethics anticipates that the high cost of technologies (PES, gene editing, brain chips) will restrict access to the wealthy, widening socioeconomic divides and transgressing distributive justice (Chin et al., 2025b). In a similar vein, Islamic bioethics emphasize principles of justice (‘*adl*) and social solidarity (Padela, 2013). The high cost is feared to perpetuate systemic disadvantages and create a new social hierarchy between the “enhanced” and the “unenhanced,” which directly contravenes Islamic ideals of social equity (Padela, 2013). Hence, there is a strong convergence with both frameworks agreeing that the prohibitive costs of enhancement technologies violate fundamental principles of equity and justice, potentially leading to elitism and discrimination.

The survey responses strongly point to the necessity of regulatory governance. Respondents not only voiced concern over the ethical implications but also advocated for government involvement to ensure safe, fair, and culturally sensitive implementation. These findings suggest that a Malaysian regulatory policy on human enhancement technologies should adopt a three-pronged governance approach as indicated in Table 3, which encompasses (i) bioethical safeguards, (ii) public engagement and education, and (iii) economic accessibility. Such a proposed three-pronged governance approach can be realistically implemented through Malaysia's existing institutional architecture (Isa et al., 2015; Saidun, 2024). At the national level, the National Bioethics Council (NBC) serves as a multidisciplinary advisory body that could facilitate dialogue between policymakers, doctors, scientists, bioethicists, and religious leaders on emerging human enhancement technologies. In parallel, the Department of Islamic Development Malaysia (JAKIM) and the State Fatwa Committees play crucial roles in providing *Shari'ah*-compliant guidance and issuing fatwas that align biomedical innovation with Islamic ethical norms (Isa et al., 2015; Saidun, 2024). Collaboration among these institutions would ensure that policy formulation remains both scientifically informed and religiously grounded, thereby enhancing the legitimacy and public acceptance of future regulatory measures. This would align with international guidance from the WHO's 2021 report on human genome editing, which calls for nation-specific regulatory pathways that address public trust, safety, and ethical diversity. Finally, the expectation among 261 (45.4%) respondents that human enhancement technology would influence decisions about childbearing due to financial burden indicates that respondents are already forecasting downstream socioeconomic impacts of human enhancement technologies. These concerns underscore the urgency of anticipatory governance. As brain chip and reprogenetic technologies become increasingly affordable and commercialized worldwide, Malaysia will need to formulate proactive rather than reactive policies. Perhaps, a deliberative bioethics approach could facilitate complex policy-making decisions on human enhancement technologies within the Malaysian context, by using structured, public dialogue to address complex ethical issues in healthcare and biomedical science (Kim et al., 2009; Meagher and Lee, 2016; Gutmann and Wagner, 2017; Dzur and Levin, 2007). Indeed, the UNESCO Universal Declaration on Bioethics and Human Rights includes a framework for deliberative bioethics (UNESCO, 2005). This approach is in fact a form of deliberative democracy, based on inclusive participation and reasoned argumentation to reach public consensus and informed policy decisions on key issues such as resource allocation and criteria for clinical application (Kim et al., 2009; Meagher and Lee, 2016; Gutmann and Wagner, 2017; Dzur and Levin, 2007). The process is initiated by gathering together different segments of the public, including non-experts, to learn more about a specific bioethics topic by engaging with experts and through knowledge exchange, in order to develop a more informed understanding of the topic at hand. This is subsequently followed by reasoned argumentation, which focuses on reasoned discussion and the exchange of arguments rather than simple debate to build consensus or compromise. Finally, informed recommendations are put forward for policy decision-making via interactive dialogue that bridges expert and public perspectives. This approach thus comprehensively integrates different types of expertise from different segments of society, thereby ensuring that decisions affecting healthcare services are made with public input (Kim et al., 2009; Meagher and Lee, 2016; Gutmann and Wagner, 2017; Dzur and Levin, 2007). Additionally, scenario-based simulations and public foresight workshops could be conducted, integrating the views of policymakers, religious leaders, bioethicists, and the general public. These platforms would enable society to co-develop ethical guardrails and avoid the pitfalls of techno-authoritarianism or unregulated commercialization. Such participatory governance frameworks have been successfully piloted in the EU through the “Horizon Scanning” initiatives (Michels et al., 2024) and could be localized in the Malaysian context.

**Table 3:** Three-pronged governance approach.

**No**.	**Governance approach**	**Details**
1.	Bioethical safeguards	Involving the National Bioethics Council, religious bodies, and independent review boards.
2.	Public engagement and education	To publicize information about risks, benefits, and ethical dilemmas of human enhancement technologies.
3.	Economic accessibility	Via tiered healthcare subsidies, particularly for the B40 (bottom 40% income) group.

## Conclusion

The public opinion of Malaysian Muslims regarding human enhancement technologies shows a mixture of skepticism, apprehension, and cautious support for therapeutic applications, particularly concerning ethical and religious implications. While there is significant backing for public healthcare subsidies and a cautious approach towards reprogenetic technologies like gene editing, the overwhelming majority of the respondents remain wary of altering fundamental aspects of human nature. However, it must be noted that a major limitation of this study is that it focused exclusively on Muslim perspectives on human enhancement technologies. This is because there are insufficient numbers of non-Muslim students/staff at the campus of the International Islamic University of Malaysia who can participate in this study. Nevertheless, Malaysia is a multi-racial and multi-religious country, so it is essential that diverse perspectives from the various ethnic and religious communities within the country are balanced, to ensure a representative and informed deliberation on human enhancement technologies. Any move towards the legalization of such technologies must be accompanied by comprehensive ethical frameworks, inclusive public consultations that encompass different strata of Malaysia's diverse multi-religious and multi-racial society, together with stringent regulatory oversight to ensure societal acceptance and ethical integrity. A topic for future research would be to explore how bioethics education and literacy (Levitt, 2003) can shape attitudes toward human enhancement technologies. For example, a survey study can be conducted to compare the responses of medical undergraduates who have completed a bioethics module vs. those who have not yet received the module. Yet another interesting area of future research would be to conduct a similar survey study on non-Muslim respondents in Malaysia, in particular the major religious minority communities within the country, such as Christians, Buddhists, and Hindus, to evaluate how their attitudes toward human enhancement technologies differ and contrast with Muslims.

## Data Availability

The original contributions presented in the study are included in the article/Supplementary material, further inquiries can be directed to the corresponding authors.
