# Supplementary material for: Human cognitive enhancement and reprogenetic technologies in Malaysia – A survey study of local Muslim undergraduate students' viewpoints
Source: Front Sociol. 2026 Jan 15;10:1701007. doi: 10.3389/fsoc.2025.1701007 (PMC12853642; doi:10.3389/fsoc.2025.1701007)
Supplement: Supplementary file 1 [file Supplementary_file_1.pdf]

Brief introduction

With the drastic decline in fertility rates worldwide and in Malaysia, parents tend to place more hope and expectations on their fewer children, investing more time, money and effort per child.

This has resulted in a more competitive educational system, which further motivates prospective parents to use new technologies for enhancing the intelligence and academic performance of their children, as well as non-diseases traits such as height and skin color. These include:

(i) DNA testing of IVF embryos (without genetic modification)

(ii) Gene editing of IVF embryos

(iii) Brain chip implants for enhancing intelligence and memory

The purpose of this survey study is to analyze the acceptability of these new human enhancement technologies to Muslim respondents and their perceptions of potential social problems associated with these new technologies, such as widening the gap between the rich and the poor.

Pengenalan ringkas

Dengan penurunan drastik dalam kadar kesuburan di seluruh dunia dan di Malaysia, ibu bapa cenderung untuk meletakkan lebih banyak harapan dan harapan kepada anak-anak mereka yang lebih sedikit, melaburkan lebih banyak masa, wang dan usaha bagi setiap anak.

Ini telah menghasilkan sistem pendidikan yang lebih kompetitif, yang seterusnya mendorong bakal ibu bapa untuk menggunakan teknologi baharu untuk meningkatkan kecerdasan dan prestasi akademik anak-anak mereka, serta ciri-ciri bukan penyakit seperti ketinggian dan warna kulit. Ini termasuk:

(i) Ujian DNA embrio IVF (tanpa pengubahsuaian genetik)

(ii) Penyuntingan gen embrio IVF

(iii) Implan cip otak untuk meningkatkan kecerdasan dan ingatan

Oleh itu, tujuan kajian tinjauan ini adalah untuk menganalisis kebolehterimaan teknologi peningkatan Manusia baharu ini kepada responden Muslim dan bukan Islam, dan persepsi mereka tentang potensi masalah sosial yang berkaitan dengan teknologi baharu ini seperti melebarkan jurang antara kaya dan miskin.
