# Supplementary material for: Human cognitive enhancement and reprogenetic technologies in Malaysia – A survey study of local Muslim undergraduate students' viewpoints
Source: Front Sociol. 2026 Jan 15;10:1701007. doi: 10.3389/fsoc.2025.1701007 (PMC12853642; doi:10.3389/fsoc.2025.1701007)
Supplement: Supplementary file 2 [file Supplementary_file_2.pdf]

Personal Information / Maklumat Peribadi

- o **Age** / *Umur* __________________

- o **Gender** / *Jantina* __________________

- o **Religion (Muslim, Non-Muslim)** / *Agama (Islam, Bukan Islam)*
  ____

- o **Education Level** / *Tahap Pendidikan* __________________
  (Can write undergraduate or postgraduate student /
  Boleh menulis prasiswazah atau pascasiswazah)

- o **Field of Study or Work** / *Bidang Pengajian atau Pekerjaan*
  ____

Survey Questions / Soalan Kajiselidik

1. **Embryo Screening** (No Genetic Modification) - Do you think it is acceptable to use DNA testing for choosing human IVF embryos based on traits like intelligence or physical appearance (height or skin colour), even when there is no health reason to do so? (For example, selecting an embryo with genes for high IQ instead of just letting nature decide).
Pemeriksaan Embrio *(Tiada Pengubahsuaian Genetik) - Adakah anda rasa dibenarkan menggunakan ujian DNA untuk memilih embrio IVF manusia berdasarkan ciri-ciri seperti kecerdasan atau penampilan fizikal (ketinggian atau warna kulit), walaupun tiada sebab kesihatan untuk berbuat demikian? (Sebagai contoh, memilih embrio dengan gen untuk IQ tinggi dan bukannya membiarkan alam semula jadi membuat keputusan).*

[ ] Strongly Agree / Sangat Bersetuju

[ ] Agree / Bersetuju

[ ] Neutral / Neutral

[ ] Disagree / Tidak Bersetuju

[ ] Strongly Disagree / Sangat Tidak Setuju

2. **Gene Editing** - Do you think it is acceptable to change an embryo's genes to improve traits like intelligence, knowing these changes will be passed on to future generations? (For example, editing genes to make a child smarter, which would affect their future children as well).
Penyuntingan Gen *- Adakah anda fikir tidak mengapa untuk menukar gen embrio untuk meningkatkan ciri-ciri seperti kecerdasan, mengetahui perubahan ini akan diturunkan kepada generasi akan datang? (Sebagai contoh, mengedit gen untuk menjadikan kanak-kanak lebih pintar, yang akan menjejaskan anak-anak masa depan mereka juga).*

[ ] Strongly Agree / Sangat Bersetuju

[ ] Agree / Bersetuju

[ ] Neutral / Neutral

[ ] Disagree / Tidak Bersetuju

[ ] Strongly Disagree / Sangat Tidak Setuju

3. **<u>Brain Chips</u>** - Do you think it is acceptable to use brain implants like a microchip to make someone smarter, even if they don't have any medical problems? (For example, implanting a chip to enhance memory or learning skills in a healthy person).
Cip Otak *- Adakah anda fikir dibenar menggunakan implan otak seperti mikrocip, untuk menjadikan seseorang lebih bijak, walaupun mereka tidak mempunyai sebarang masalah perubatan? (Contohnya, menanam cip untuk meningkatkan ingatan atau kemahiran belajar pada orang yang sihat).*

[ ] Strongly Agree / Sangat Bersetuju

[ ] Agree / Bersetuju

[ ] Neutral / Neutral

[ ] Disagree / Tidak Bersetuju

[ ] Strongly Disagree / Sangat Tidak Setuju

4. **Religious Concerns** - Should the Malaysian government ban technologies like gene editing for Muslims due to Shari'ah concerns?
Kebimbangan Agama *- Patutkah kerajaan mengharamkan teknologi seperti penyuntingan gen untuk umat Islam kerana kebimbangan Syariah?*

[ ] Strongly Agree / Sangat Bersetuju

[ ] Agree / Bersetuju

[ ] Neutral / Neutral

[ ] Disagree / Tidak Bersetuju

[ ] Strongly Disagree / Sangat Tidak Setuju

5. Briefly **write down** any religious or ethical objections you may have against these new human enhancement technologies:
Tuliskan *secara ringkas sebarang bantahan agama atau etika yang mungkin anda miliki terhadap teknologi peningkatan Manusia baharu ini:*

____

____

____

____

____

____

____

6. **Social Inequalities** - Do you think that uptake of these human enhancement technologies will increase social inequalities, because only the rich can afford these? Will this further widen the gap between the rich and the poor?
Ketaksamaan Sosial *- Adakah anda berpendapat bahawa penggunaan teknologi peningkatan Manusia ini akan meningkatkan ketidaksamaan sosial, kerana hanya orang kaya yang mampu membelinya? Adakah ini akan melebarkan lagi jurang antara yang kaya dan yang miskin?*

[ ] Strongly Agree / Sangat Bersetuju

[ ] Agree / Bersetuju

[ ] Neutral / Neutral

[ ] Disagree / Tidak Bersetuju

[ ] Strongly Disagree / Sangat Tidak Setuju

7. **Racial Disparities** - Do you expect that there will be disparities in the uptake of these human enhancement technologies by the different races and ethnic groups in Malaysia, which might widen the socioeconomic gap between the different races?
Ketidaksamaan Kaum *- Adakah anda menjangkakan bahawa akan wujud jurang perbezaan dalam penggunaan teknologi peningkatan Manusia ini oleh pelbagai kaum dan kumpulan etnik di Malaysia, yang mungkin melebarkan jurang sosioenomik antara kaum yang berbeza?*

[ ] Strongly Agree / Sangat Bersetuju

[ ] Agree / Bersetuju

[ ] Neutral / Neutral

[ ] Disagree / Tidak Bersetuju

[ ] Strongly Disagree / Sangat Tidak Setuju

8. **Legal Approval** - Do you think that the Malaysian government should approve and permit the uptake of these human enhancement technologies in the country?
Kelulusan Undang-undang *- Adakah anda berpendapat bahawa kerajaan Malaysia harus meluluskan dan membenarkan penggunaan teknologi peningkatan Manusia ini di negara ini?*

[ ] Strongly Agree / Sangat Bersetuju

[ ] Agree / Bersetuju

[ ] Neutral / Neutral

[ ] Disagree / Tidak Bersetuju

[ ] Strongly Disagree / Sangat Tidak Setuju

9. **Permitting or banning according to religion** - Do you think that it would be fair for the Malaysian government to ban the uptake of some of these human enhancement technologies by Muslims due to violation of Shariah law (For example, gene editing for enhancement would be alteration of Allah's creation), while permitting uptake by non-Muslims?

Membenarkan atau mengharamkan mengikut agama *- Adakah anda fikir adalah adil bagi kerajaan Malaysia untuk mengharamkan penggunaan beberapa teknologi peningkatan Manusia ini oleh orang Islam kerana melanggar undang-undang Syariah (contohnya penyuntingan gen untuk penambahbaikan akan menjadi pengubahsuaian ciptaan Allah), sambil membenarkan pengambilan oleh bukan Islam?*

[ ] Strongly Agree / Sangat Bersetuju

[ ] Agree / Bersetuju

[ ] Neutral / Neutral

[ ] Disagree / Tidak Bersetuju

[ ] Strongly Disagree / Sangat Tidak Setuju

10. **Public Subsidies** - If the Malaysian government legally permits the uptake of these human enhancement technologies, should there be public healthcare subsidies for poorer people to utilize these technologies?
Subsidi Awam *- Jika kerajaan Malaysia secara sah membenarkan penggunaan teknologi peningkatan manusia ini, adakah perlu ada subsidi penjagaan kesihatan awam untuk golongan miskin menggunakan teknologi ini?*

[ ] Strongly Agree / Sangat Bersetuju

[ ] Agree / Bersetuju

[ ] Neutral / Neutral

[ ] Disagree / Tidak Bersetuju

[ ] Strongly Disagree / Sangat Tidak Setuju

11. **Social Pressure** - If you see some of your friends and relatives using such human enhancement technologies to have smarter and more beautiful kids, will you be under social pressure to do the same?
Tekanan Sosial *- Jika anda melihat beberapa rakan dan saudara-mara anda menggunakan teknologi peningkatan Manusia sedemikian untuk mempunyai anak yang lebih pintar dan cantik, adakah anda akan berada di bawah tekanan sosial untuk melakukan perkara yang sama?*

[ ] Strongly Agree / Sangat Bersetuju

[ ] Agree / Bersetuju

[ ] Neutral / Neutral

[ ] Disagree / Tidak Bersetuju

[ ] Strongly Disagree / Sangat Tidak Setuju

12. **Fewer Children due to Financial Burden** - If you feel obliged to spend so much money on such human enhancement technologies for your kids, will you plan to have fewer kids?
Lebih Sedikit Kanak-kanak kerana Beban Kewangan - Jika anda berasa bertanggungjawab untuk membelanjakan begitu banyak wang untuk teknologi peningkatan manusia sedemikian untuk anak-anak anda, adakah anda merancang untuk mempunyai lebih sedikit anak-anak?

[ ] Strongly Agree / Sangat Bersetuju

[ ] Agree / Bersetuju

[ ] Neutral / Neutral

[ ] Disagree / Tidak Bersetuju

[ ] Strongly Disagree / Sangat Tidak Setuju
